# Consolidated bioprocessing for bioethanol production by metabolically engineered *Bacillus subtilis* strains

**DOI:** 10.1038/s41598-021-92627-9

**Published:** 2021-07-02

**Authors:** Fatemeh Maleki, Mohammad Changizian, Narges Zolfaghari, Sarah Rajaei, Kambiz Akbari Noghabi, Hossein Shahbani Zahiri

**Affiliations:** Department of Energy and Environmental Biotechnology, National Institute of Genetic Engineering and Biotechnology (NIGEB), Tehran, Iran

**Keywords:** Industrial microbiology, Metabolic engineering, Molecular engineering

## Abstract

Bioethanol produced by fermentative microorganisms is regarded as an alternative to fossil fuel. Bioethanol to be used as a viable energy source must be produced cost-effectively by removing expense-intensive steps such as the enzymatic hydrolysis of substrate. Consolidated bioprocessing (CBP) is believed to be a practical solution combining saccharification and fermentation in a single step catalyzed by a microorganism. *Bacillus subtills* with innate ability to grow on a diversity of carbohydrates seems promising for affordable CBP bioethanol production using renewable plant biomass and wastes. In this study, the genes encoding alcohol dehydrogenase from *Z. mobilis* (*adh*_*Z*_) and *S. cerevisiae* (*adh*_*S*_) were each used with *Z. mobilis* pyruvate decarboxylase gene (*pdc*_*Z*_) to create ethanologenic operons in a lactate-deficient (Δ*ldh*) *B. subtilis* resulting in NZ and NZS strains, respectively. The *S. cerevisiae adh*_*S*_ caused significantly more ethanol production by NZS and therefore was used to make two other operons including one with double copies of both *pdc*_*Z*_ and *adh*_*S*_ and the other with a single *pdc*_*Z*_ but double *adh*_*S*_ genes expressed in N(ZS)2 and NZS2 strains, respectively. In addition, two fusion genes were constructed with *pdc*_*Z*_ and *adh*_*S*_ in alternate orientations and used for ethanol production by the harboring strains namely NZ:S and NS:Z, respectively. While the increase of gene dosage was not associated with elevated carbon flow for ethanol production, the fusion gene *adh*_S_:*pdc*_Z_ resulted in a more than two times increase of productivity by strain NS:Z as compared with NZS during 48 h fermentation. The CBP ethanol production by NZS and NS:Z using potatoes resulted in 16.3 g/L and 21.5 g/L ethanol during 96 h fermentation, respectively. For the first time in this study, *B. subtilis* was successfully used for CBP ethanol production with *S. cerevisiae* alcohol dehydrogenase*.* The results of the study provide insights on the potentials of *B. subtilis* for affordable bioethanol production from inexpensive plant biomass and wastes. However, the potentials need to be improved by metabolic and process engineering for higher yields of ethanol production and plant biomass utilization.

## Introduction

There has been a growing interest in using agricultural wastes and by-products as abundant, non-expensive, and non-food feedstocks for bioethanol production. Plant biomass is mainly composed of cellulose and starch that need to be hydrolyzed into fermentable sugars before being used for ethanol production^1^. Natural ethanologenic microorganisms like *Saccharomyces cerevisiae* and *Zymomonas mobilis* can utilize just a few simple sugars but, of course, not starch and other polysaccharides. Therefore, the polymeric plant biomass needs to be converted into consumable sugars for fermentation by the microorganisms. In order to reduce the costs, it is desirable to obtain an ethanologenic strain that can consume polymeric carbohydrates on its own to integrate the saccharification and fermentation in a consolidated process. For this purpose, several attempts have been made to transform ethanologenic strains by the expression of exogenous enzymes and pathways to broaden the range of substrates they can utilize for ethanol production^2–4^. However, there are still drawbacks as to the efficiency of ethanologenic microorganisms for the production and secretion of exogenous enzymes as well as their tolerance against the toxicity and inhibitory effects of biomass hydrolysates. On the other hand, it is quite tempting to convert non-ethanologenic microorganisms that are able to grow on a wide range of carbon sources into ethanol producers^5,6^. In this regard, gram-positive bacteria seem to be appropriate candidates due to having beneficial properties such as the ability to survive at high temperature and low pH conditions, and tolerance of high concentrations of sugar, salt, and ethanol. *Bacillus subtilis* is a gram-positive bacterium that is generally recognized as safe (GRAS) and is amenable to genetic manipulation as well^7^. This bacterium with an optimum temperature of 37 °C can grow at temperatures up to 50 °C and ferments many carbohydrates from mono-, di-, oligo-, and polysaccharides. In this regard, the ability of *B. subtilis* to utilize starch, xylan, galactan, pullulan, arabinan, rhamnogalacturonan, and pectin is quite interesting when it comes to converting plant biomass wastes into biotechnological products with added value. The capability of *B. subtilis* to survive on different carbohydrates is largely due to the production and efficient secretion of various hydrolytic enzymes by this bacterium. Therefore, it is quite intriguing to take advantage of the ability to extend the substrate range of *B. subtilis* by the expression of exogenous genes encoding novel enzymes required for the complete degradation of plant biomass^8^.

**Figure 1:**
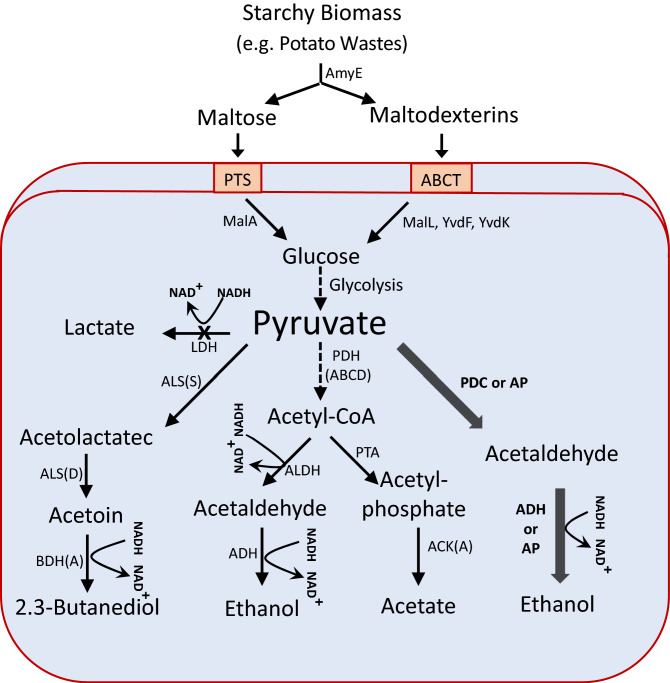
Schematic representation of *B. subtilis* fermentation metabolism and ethanol production from potatoes using the engineered heterologous pathways. The thin arrows indicate the intrinsic metabolic pathways and the thick arrows point to the engineered pathway composed of either PDC and ADH or the bifunctional AP fusion. *AmyE* extracellular amylase, *PTS* phosphotransferase system, *MalA* phospho-α-1,4-glucosidase, *ABCT* ABC transporter, *YvdF* maltogenic amylase, *YvdK* maltose phosphorylase, *MalL* glucosidase, *LDH* lactate dehydrogenase, *ALS* acetolactate synthase; *BDH* butanediol dehydrogenase, *PDH* pyruvate dehydrogenase, *ALDH* acetaldehyde dehydrogenase, *PTA* phosphotransacetylase, *ACK* acetate kinase, *PDC* pyruvate decarboxylase, *ADH* alcohol dehydrogenase, *AP* bifunctional AHD:PDC fusion.

However, for bioethanol production, *B. subtilis* needs to be improved by genetic manipulation and pathway engineering as the inherent capacity of this bacterium for ethanol production is quite negligible. The only successful attempt to develop an ethanologenic strain of *B. subtilis* has been reported by Romero et al.^6^. They managed to create the strain by engineering an exogenous ethanol pathway using heterologous expression of *Z. mobilis* genes encoding pyruvate decarboxylase (*pdc*) and alcohol dehydrogenase (*adhB*). In addition, they had to knock out the native genes encoding lactate dehydrogenase (*ldh*) and acetolactate synthase (*alsS*) to obstruct lactate and 2,3-butanediol production, respectively, as major fermentation products of *B. subtilis*. To this end, the *ldh* was disrupted by the insertion of *Z. mobilis pdc* and *adhB* genes; and *alsS* was knocked out by the insertion of *E. coli udhA* encoding a transhydrogenase to balance NADH/NADPH ratio. By the redirection of the fermentative metabolism, the resulting strain was able to produce 8.9 g/L ethanol from 20 g/L glucose during 9 days of fermentation under nonaerated conditions. Despite the interesting results, the ethanol titer needs to be improved significantly to become economically viable. A positive point of *B. subtilis* compared to natural ethanologenic strains such as *S. cerevisiae* is that the bacterium can utilize polysaccharides, and thus it might be used in CBP systems for affordable bioethanol production from plant biomass without the need for enzymatic pretreatments. Plant biomass polysaccharides, including cellulose and starch, comprise the most abundant renewable resource of organic matter in the world. The invaluable resource holds strong potentials to be used as feedstock for the production of biofuels, fine chemicals, and other materials. However, the polysaccharides, before being used in many applications, usually require to be converted into fermentable sugars by enzymatic treatments incurring heavy expenses on the whole process. A plausible solution to this challenge is to combine the saccharification and fermentation into a single step by CBP in which a microorganism is responsible to catalyze the whole process. With regard to this point, the current study was conducted to evaluate the potential of *B. subtilis* for bioethanol production from untreated potatoes in a consolidated bioprocess (Fig. 1). For this purpose, lactate production as a significant rival pathway had to be eliminated. The ethanologenic *B. subtilis* strains were engineered using the genes encoding *Z. mobilis* pyruvate decarboxylase (*pdc*) and the alcohol dehydrogenase of *Z. mobilis* (*adhB*) and *S. cerevisiae* (*adh*I). Also, the influence of additional copies of *pdc* and *adh*, as well as their fusions encoding bifunctional enzymes, were analyzed on bioethanol production by *B. subtilis*. The resulting strains were investigated for ethanol production on glucose as well as for CBP ethanol production on potatoes.

## Materials and methods

### Strains

The strains, plasmids, and primers used in this study are listed in Table 1. The strains of *Zymomonas mobilis* 1718 and *Saccharomyces cerevisiae* 5080 were purchased from PTCC (Tehran, Iran). *Z. mobilis* was cultured in a medium composed of 1% Bacto peptone, 1% yeast extract, 2% glucose at 30 °C. *S. cerevisiae* was grown at 26 °C in a culture medium with 1% glucose, 0.5% peptone, 0.3% yeast extract, 0.3% malt extract. *Escherichia coli* DH5α and *B. subtilis* WB600 were available from the laboratory stock and routinely cultured on LB (Luria–Bertani) medium (1% tryptone, 0.5% yeast extract, and 1% NaCl) at 37 °C. *E. coli* DH5α was used as a host for the construction of recombinant plasmids containing ethanologenic operons. The transformation of *E. coli* DH5α was performed by the heat shock method and the transformants were selected on LB agar plates with ampicillin according to standard protocols^9^. *B. subtilis* WB600 was used for the creation of ethanologenic strains by deletion of *ldh* (encoding lactate dehydrogenase) and transformation of the resulting strain (*B. subtilis* WBN) with recombinant plasmids using natural transformation according to Anagnostopoulos and Spizizen^10^.

**Table 1:** list of strains, plasmids, and primers used in this study.

Strains	Description	Source
*E.coli* DH5α	F^−^ φ80*dlacZ*ΔM15 Δ(*lacZYA-argF*) U169 *deoR recA1 endA1 hsdR17*(r_K_^−^, m ^+^) *phoA supE44* λ^−^ *thi-1 gyrA96 relA1*	Invitrogen
*Z. mobilis*	Ethanologenic strain	PTCC 1718
*S. cerevisiae*	Ethanologenic strain	PTCC 5080
*B.* *subtilis* WB600	*trp*C2 *npr*E *apr*E *epr bpr mpr npr*B; Em^r^	^11^
*B.* *subtilis* WBN	*Bacillus subtilis* WB600, *ldh*::pDH*tldh*	This study
*B.* *subtilis* NC	WBN harboring pHY300PLK	This study
*B.* *subtilis* NZ	WBN harboring pHY*pdc*_*Z*_*-adh*_*Z*_	This study
*B.* *subtilis* NZS	WBN harboring pHY*pdc*_*Z*_*-adh*_*S*_	This study
*B.* *subtilis* NZS2	WBN harboring pHY*pdc*_*Z*_*-*(*adh*_*S*_)_2_	This study
*B.* *subtilis* N(ZS)2	WBN harboring pHY(*pdc*_*Z*_*-adh*_*S*_)_2_	This study
*B.* *subtilis* NZ:S	WBN harboring pHY*pdc*_Z_*:adh*_S_	This study
*B.* *subtilis* NS:Z	WBN harboring pHY*adh*_S_:*pdc*_Z_	This study

Plasmids	Description	Source
pHY300PLK	Shuttle vector, Tet and Amp resistant	^12^
pHY*pdc*_*Z*_*-adh*_*Z*_	pHY300PLK with *pdc* and *adhII* of *Z. mobilis*	This study
pHY*pdc*_*Z*_*-adh*_*S*_	pHY300PLK with Z. *mobilis pdc* and *S. cerevisiae adhI*	This study
pHY*pdc*_*Z*_*-*(*adh*_*S*_)_2_	pHY300PLK with Z. *mobilis pdc* and two copies of *S. cerevisiae adhI*	This study
pHY(*pdc*_*Z*_*-adh*_*S*_)_2_	pHY300PLK with two copies of both *Z. mobilis pdc* and *S. cerevisiae adhI*	This study
pHY*adh*_S_:*pdc*_Z_	pHY300PLK with *adh*_S_:*pdc*_Z_ fusion gene	This study
pHY*pdc*_Z_:*adh*_S_	pHY300PLK with *pdc*_Z_:*adh*_S_ fusion gene	This study
pDH88	Integration vector, chloramphenicol resistant	^13^
pDH*tldh*	pHD88 with a 325 bp fragment from within *B. subtilis ldh*	This study

Primers	Sequence	Restriction enzymes
*pdc*_Z_-F	ATTGAATTCCATATGAAGGAGGAGTAAGCAATGAGTTATACTGTCGG	*Eco*RI
*pdc*_Z_-R	ATAGGATCCCTAGAGGAGCTTGTTAACAGGCTTACG	*Bam*HI
*adh*_Z_-F	ATAGGATCCAAGGAGGGTATAGCTATGGCTTCTTCAACTTTTTATATTC	*Bam*HI
*adh*_Z_-R	ATATCTAGACATATGTCAGAAAGCGCTCAGGAAGAGTTCTTCAAC	*Xba*I
*adh*_S_-F	ATGGATCCAAAGGAGGCGATTTGATGTCTATCCCAGAAACTC	*Bam*HI
*adh*_S_-R	ATTCTAGATTAATGATGATGATGATGATGTTTAGAAGTGTCAACAAC	*Xba*I
*tldh*-F	ATAAAGCTTTCTTACGGAACATATGAAGACTGC	*Hind*III
*tldh*-R	ATATCTAGACGTGTACGTTTTGAGGCGC	*Xba*I
*dldh*-F	GTAGCTTTAATCGGAGCGGG	–
*dldh*-R	GCGACATCGTATAACGTTACTGG	–

### Construction of ethanologenic operons in pHY300PLK

The molecular biology methods such as restriction digestion, ligation, transformation, and agarose gel electrophoresis were all according to standard protocols^9^. The *pdc* and *adh* genes were obtained from *Z. mobilis* and *S. cerevisiae* by PCR. For this purpose, the overnight cultures of *Z. mobilis* and *S. cerevisiae* were harvested by centrifugation (12,000×*g*, 20 min) and used for DNA extraction using a genomic DNA purification kit (GeneAll, Korea). The purified DNA of each strain was used as a template in PCR reactions with specific primers listed in Table 1. The amplified genes were purified and digested with appropriate restriction enzymes and then ligated into pHY300PLK to make artificial operons using *Z. mobilis pdc* and *adhII* or *Z. mobilis pdc* and *S. cerevisiae adhI* under the control of *Tet* promoter. Therefore, two recombinant plasmids namely pHY*pdc*_*Z*_*-adh*_*Z*_ and pHY*pdc*_*Z*_*-adh*_*S*_ were constructed (Table 1). In addition, two other plasmids including pHY(*pdc*_*Z*_*-adh*_*S*_)_2_ with an operon containing two copies of both *pdc*_*Z*_ and *adh*_*S*_ and the other plasmid pHY*pdc*_*Z*_*-*(*adh*_*S*_)_2_ with an operon containing one copy of *pdc*_*Z*_ but two copies of *adh*_*S*_ were constructed. Also, two fusion genes were synthesized using *Z. mobilis pdc* and *S. cerevisiae adh* by in-frame cloning of the genes consecutively in pHY300PLK^12^. The genes were fused in alternate orientations while the stop nucleotides of the first gene and the start nucleotides of the second gene were deleted. Therefore, two plasmids namely pHY*pdc*_Z_:*adh*_S_ and pHY*adh*_S_:*pdc*_Z_ were constructed (Table 1). The authenticity of the resulting plasmids was confirmed by sequencing (Pishgam Company, Tehran, Iran).

### Inactivation of *ldh* in *B. subtilis* WB600

The chromosomal gene encoding lactate dehydrogenase (*ldh*) in *B. subtilis* WB600 was disrupted by homologous recombination. For this purpose, a DNA fragment of 325 bp was amplified from within the gene using primers *tldh*-F and *tldh*-R. The amplified fragment (named *tldh*) was digested with *Hin*dIII and *Xba*I, and then ligated into pDH88^13^. The resulting plasmid, pDH*tldh*, was used for the transformation of *B. subtilis* WB600. Transformants were selected on LB medium containing 5 µg/mL chloramphenicol and checked for a Δ*ldh* strain by PCR with *dldh*-F and *dldh*-R primers and sequencing. The select strain, named WBN, was used for transformation by every constructed plasmid including pHY*pdc*_*Z*_*-adh*_*Z*_, pHY*pdc*_*Z*_*-adh*_*S*_, pHY(*pdc*_*Z*_*-adh*_*S*_)_2_, pHY*pdc*_*Z*_*-*(*adh*_*S*_)_2_, pHY*adh*_*S*_:*pdc*_*Z*_, and pHY*pdc*_*Z*_:*adh*_*S*_ giving rise to strains NZ, NZS, N(ZS)2, NZS2, NS:Z, and NZ:S, respectively (Table 1).

### Fermentation conditions

For ethanol production, *B. subtilis* strains harboring the heterologous ethanologenic operons were cultured overnight at 37 °C in 2YT broth (1.6% Tryptone, 1% yeast extract, 0.5% NaCl, pH 7) containing 5 µg/mL chloramphenicol and 20 µg/mL tetracycline. The growing cells were used as inoculum to an initial optical density (OD_600 nm_) of 0.1 in 1-L flasks containing 100 mL of 2YT broth supplemented with either glucose or dried ground potatoes (DGP). The DGP was prepared using potatoes obtained from a local market. The potatoes were peeled, washed, and grated before being dried in an oven at 50 °C. The dried biomass was ground using a grinder and used in the preparation of culture mediums for ethanol production at 50, 100, and 150 g/L concentrations. The cultures were conducted at 37 °C and 180 rpm in flasks plugged with cotton balls as normal aeration (NA) conditions. Where limit aeration (LA) was required, the cotton-plugged flasks were covered with aluminum caps. For biphasic cultures, the growing cells were incubated under normal aeration for the first 48 h and then under limited aeration for another 48 h. At the end of fermentation, the residual biomass was separated by centrifugation (12,000×*g*, 20 min) and dried to obtain the weight.

### Analytical methods

The growth was monitored by the determination of the optical density of the culture medium at 600 nm. The glucose concentration was measured using a glucose oxidase kit (Pars Azmoon, Tehran, Iran). The ethanol concentration was measured by a Varian CP-3800 gas chromatograph equipped with a CP-Wax 57 CB column, a 1041 injector, and a flame ionization detector (FID). All GC analyses were performed under the same conditions as follows: Helium of high purity as the carrier gas at a flow rate of 1 mL/min, the injection port temperature of 200 °C, and the detector temperature of 250 °C. The ethanol concentration in the culture medium was calculated by a linear regression equation that was prepared using a series of standard ethanol concentrations in water. Methanol was used as an internal standard added into all samples at 2 g/L concentration.

## Results

### Inactivation of lactate dehydrogenase

The gene encoding lactate dehydrogenase (*ldh*) was disrupted by chromosomal insertion of pDH*tldh* using homologous recombination. *B. subtilis* WB600 was transformed with pDH*tldh* and the resulting transformant, named *B. subtilis* WBN, was selected on an LB plate with chloramphenicol. The authenticity of homologous recombination and disruption of *ldh* in WBN strain was confirmed by PCR using the *dldh*-F and *dldh*-R primers. The *dldh*-F was designed to anneal to WBN chromosomal DNA just upstream to *tldh* sequence while the reverse primer *dldh*-R was designed according to the *lac*I gene of pDH*tldh*. The PCR product of about 800 bp was sequenced, revealing that it contained partial sequences from both WB600 *ldh* and the *lac*I of pDH*tldh.* The result confirmed that pDH*tldh* was correctly inserted in WBN chromosome by a Campbell-like mechanism, resulting in the disruption of the *ldh* gene^14,15^.

### Comparison of *S. cerevisiae adhI *and *Z. mobilis adhB*

**Figure 2:**
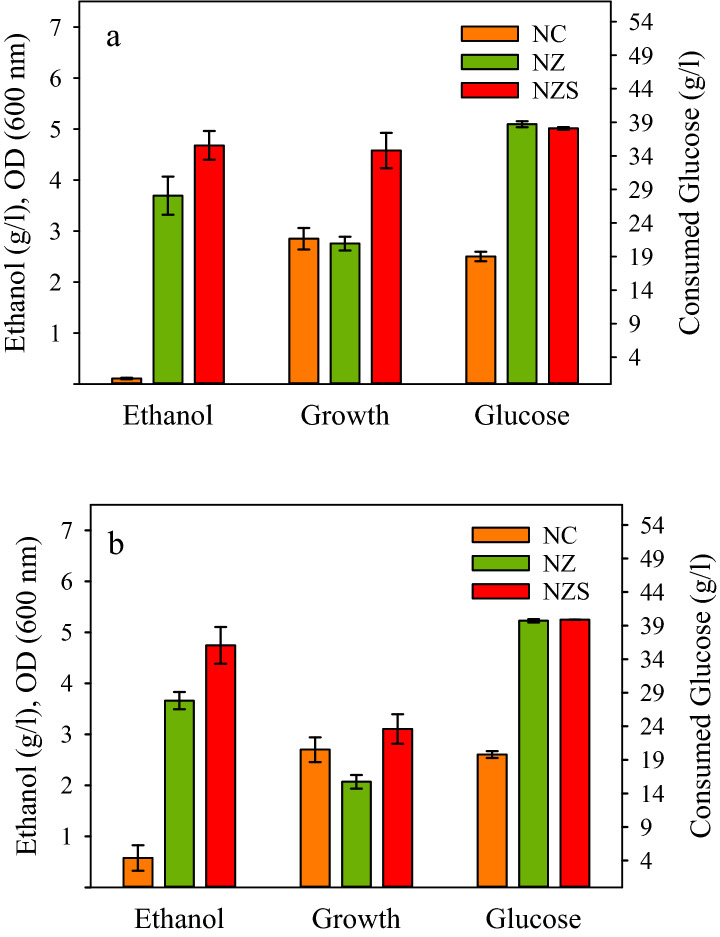
Ethanol production using strains NZ (Δ*ldh*, *pdc*_*Z*_*-adh*_*Z*_ ) and NZS (Δ*ldh*, *pdc*_*Z*_*-adh*_*S*_). The strains along with the control strain NC (Δ*ldh*) were cultured in 2YT medium containing 40 g/L glucose at 37 °C, and 180 rpm for 96 h under limited aeration conditions. Samples were taken at (**a**) 48 h, and (**b**) 96 h of fermentation, and analyzed for ethanol production, cell growth, and glucose consumption.

The efficiency of *S. cerevisiae adhI* for ethanol production was analyzed against *Z. mobilis adhB* using NZ and NZS strains. The strains were cultured in 2YT medium containing 4% glucose under limited aeration conditions. The strain NC harboring pHY300PLK was used as a control. The results showed that NZ produced 3.7 g/L ethanol but NZS was more efficient producing 4.7 g/L ethanol during 48 h of incubation. The ethanol production of NC during the same period was about 0.1 g/L (Fig. 2a). However, during the next 48 h of fermentation, ethanol concentration in the culture mediums of NZ and NZS did not change significantly but that of NC was increased to about 0.6 g/L (Fig. 2b). Although the ethanol production by normal *B. subtilis* is quite negligible, in the NC strain due to the lack of lactate production, the carbon flux could partly be allotted to ethanol production. The growth of NZ and NZS did not seem to be adversely influenced by the synthetic ethanol pathways. Both strains efficiently utilized more than 90% of initial glucose during 48 h of fermentation. In contrast, NC just consumed less than 50% of the added glucose. Given the comparable growth of the strains, the higher glucose consumption of NZ and NZS can be attributed to the function of the exogenous ethanol pathways (Fig. 2). The results showed that *adh*_*S*_ was remarkably more efficient than *adh*_*Z*_ resulting in 30% more ethanol production by *B. subtilis*.

### Ethanol production by NZS under various aeration conditions

**Figure 3:**
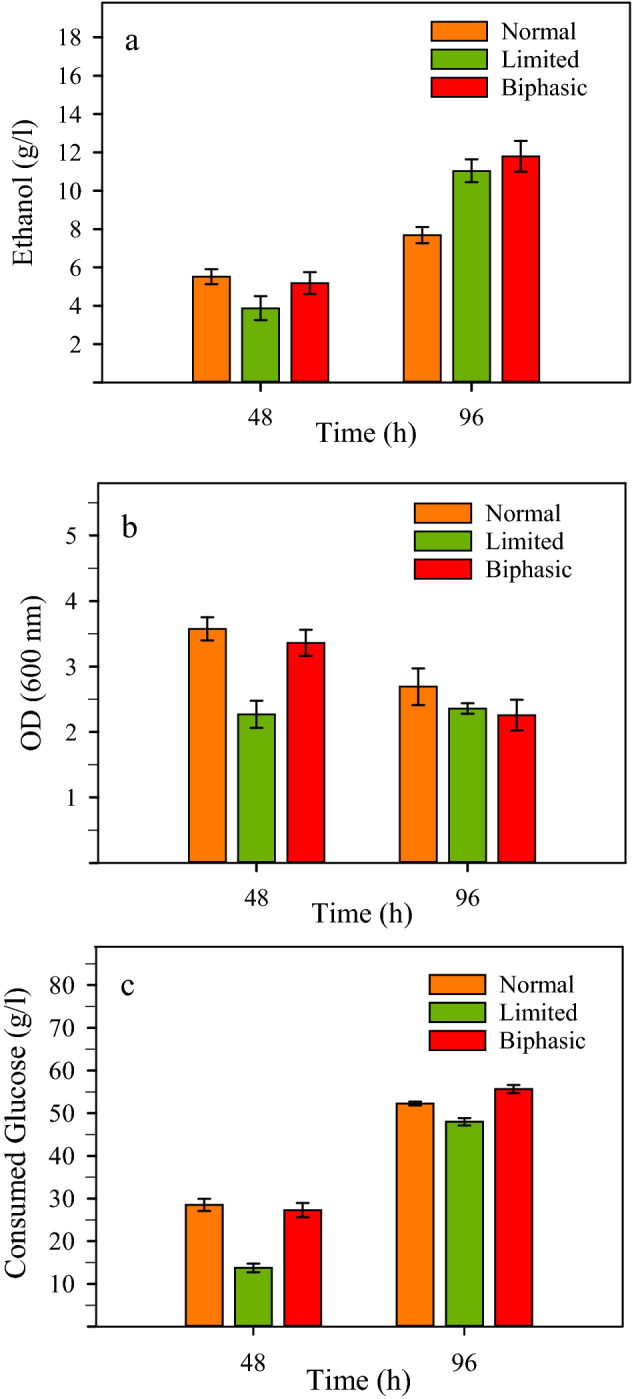
Ethanol production using *B. subtilis* NZS (Δ*ldh*, *pdc*_*Z*_*-adh*_*S*_) under various aeration conditions. Cultures were conducted for 96 h in a shaking incubator at 37 °C and 180 rpm under the normal, limited, and biphasic aeration conditions. Samples were taken at 48 h and 96 h of fermentation and analyzed for: (**a**) ethanol production, (**b**) cell growth, and (**c**) glucose consumption.

The effect of aeration on growth and ethanol production was studied under normal, limited, and biphasic aeration conditions using the strain NZS with 60 g/L glucose. Interestingly, *B. subtilis* NZS was able to produce ethanol even under the high oxygen transfer rate of normal aeration conditions (Fig. 3). However, the ethanol accumulation under such conditions was less than half of those obtained under limited and biphasic aeration. The results showed that under high aeration, much of the added glucose was consumed for growth (Fig. 3). The ethanol concentration at the end of fermentation was 7.7 g/L and the ethanol production yield from the consumed glucose was merely about 29% of the theoretical maximum. Under the limited aeration conditions, the growth was lowered providing the ethanol pathway with the chance to channel more of the carbon flow into ethanol production. The ethanol concentration was about 11 g/L after 96 h and the ethanol production yield was improved to about 45%. When cultures were conducted under the biphasic aeration conditions, the overall glucose consumption was higher than other aeration conditions, and the ethanol production was slightly improved to about 11.8 g/L. However, the ethanol production yield was about 42% of the theoretical maximum indicating that the synthetic ethanol pathway at the existing expression level was not so efficient as to appropriate a larger portion of the carbon metabolism.

### Combinatorial effects of aeration, temperature, and shaking on ethanol production

**Figure 4:**
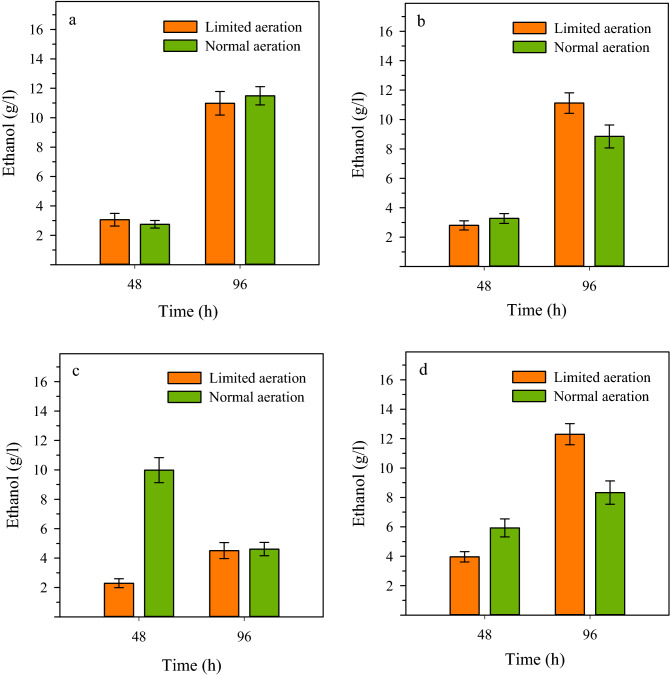
The combinatorial influence of aeration, temperature, and shaking conditions on ethanol production by strain NZS (Δ*ldh*, *pdc*_*Z*_*-adh*_*S*_). Cultures with limited and normal aeration were incubated at: (**a**) 30 °C, 120 rpm; (**b**) 30 °C, 180 rpm; (**c**) 37 °C, 120 rpm; and (**d**) 37 °C, 180 rpm. Samples obtained at 48 h and 96 h of fermentation were analyzed for ethanol concentration.

The ethanol production by NZS was evaluated in cultures with 50 g/L glucose under limited and normal aeration conditions at two temperatures of 30 and 37 °C and two shaking rates of 120 and 180 rpm (Fig. 4). The results showed that at 30 °C and 120 rpm, there was no significant difference in ethanol production with either limited or normal aeration resulting in about 11 g/L ethanol accumulation after 96 h (Fig. 4a). At the same temperature of 30 °C but elevated shaking rate of 180 rpm, growth under the limited aeration conditions resulted in a significantly higher ethanol accumulation of 11 g/L against 8.9 g/L of the normal aeration (Fig. 4b). The ethanol accumulation at 37 °C was more significantly affected by shaking rate and aeration conditions so that a high ethanol concentration of 10 g/L was accumulated just during 48 h of incubation at 120 rpm under normal aeration conditions, while only 2.3 g/L of ethanol was produced with limited aeration during the same incubation time (Fig. 4c). Finally, the ethanol production by NZS was studied at 37 °C and 180 rpm. The results showed that these culture conditions were favorable with the limited aeration resulting in an ethanol concentration of about 12.3 g/L. However, a rather high amount of ethanol (8.3 g/L) could also be produced with the normal aeration, (Fig. 4d). The results presented in Fig. 4 indicate that aeration, shaking and temperature exert a combinatorial effect on the ethanol production of NZS. These parameters manage the yield and productivity of ethanol production by influencing the growth rate and metabolism of strain NZS as well as the activity of ethanologenic enzymes. As such, the highest ethanol productivity (0.21 g/L/h) was achieved under the normal aeration at 37 °C and 120 rpm but the highest yield (48% of the theoretical maximum) was obtained under limited aeration at 37 °C and 180 rpm. The figures for yield and productivity seem quite remarkable, given that just a small inoculum was used for culture mediums and the cells had to produce ethanol while growing on total glucose of 50 g/L.

### CBP ethanol production by NZS using untreated potatoes as substrate

**Figure 5:**
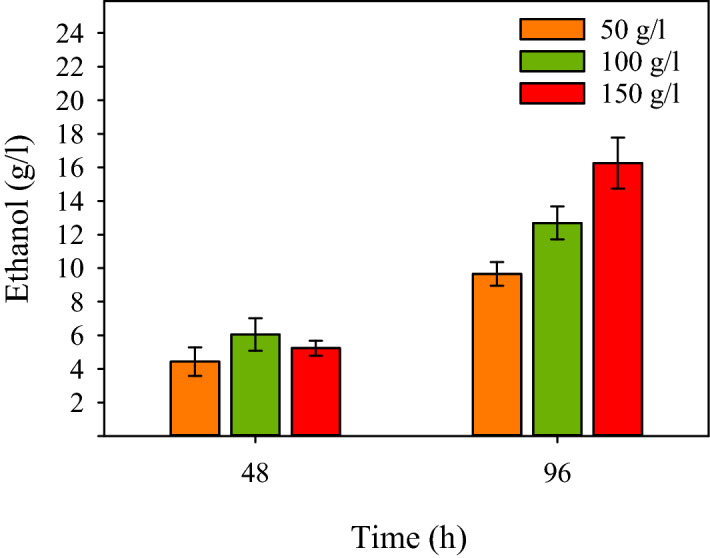
CBP ethanol production by strain NZS (Δ*ldh*, *pdc*_*Z*_*-adh*_*S*_) using potatoes as a typical starchy biomass. Cultures were conducted in 2YT medium supplemented with 50, 100, and 150 g/L dried ground potatoes (DGP) at 37 °C and 180 rpm under limited aeration using a tiny inoculum (OD_600_ 0.1) of NZS. Samples, taken after 48 h and 96 h, were analyzed for ethanol concentration in culture mediums.

With respect to the ability of *B. subtilis* to produce extracellular hydrolases, strain NZS was evaluated for CBP ethanol production using untreated potatoes as a typical starchy substrate. The results showed that NZS could grow on all tested concentrations of DGP using its native hydrolysis capacity and produced ethanol by CBP (Fig. 5). After 96 h of fermentation, the concentration of ethanol in the culture mediums with 50, 100, and 150 g/L DGP was 9.6 g/L, 12.7 g/L, and 16.3 g/L while about 26 g, 45 g, and 70 g of the initial DGP, respectively, was solubilized (data not shown). The yield of ethanol production using 50, 100, and 150 g/L DGP was about 65%, 50%, and 41% of the theoretical maximum, estimated roughly based on the solubilized biomass.

### Effects of gene copy number and fusions on ethanol production

**Figure 6:**
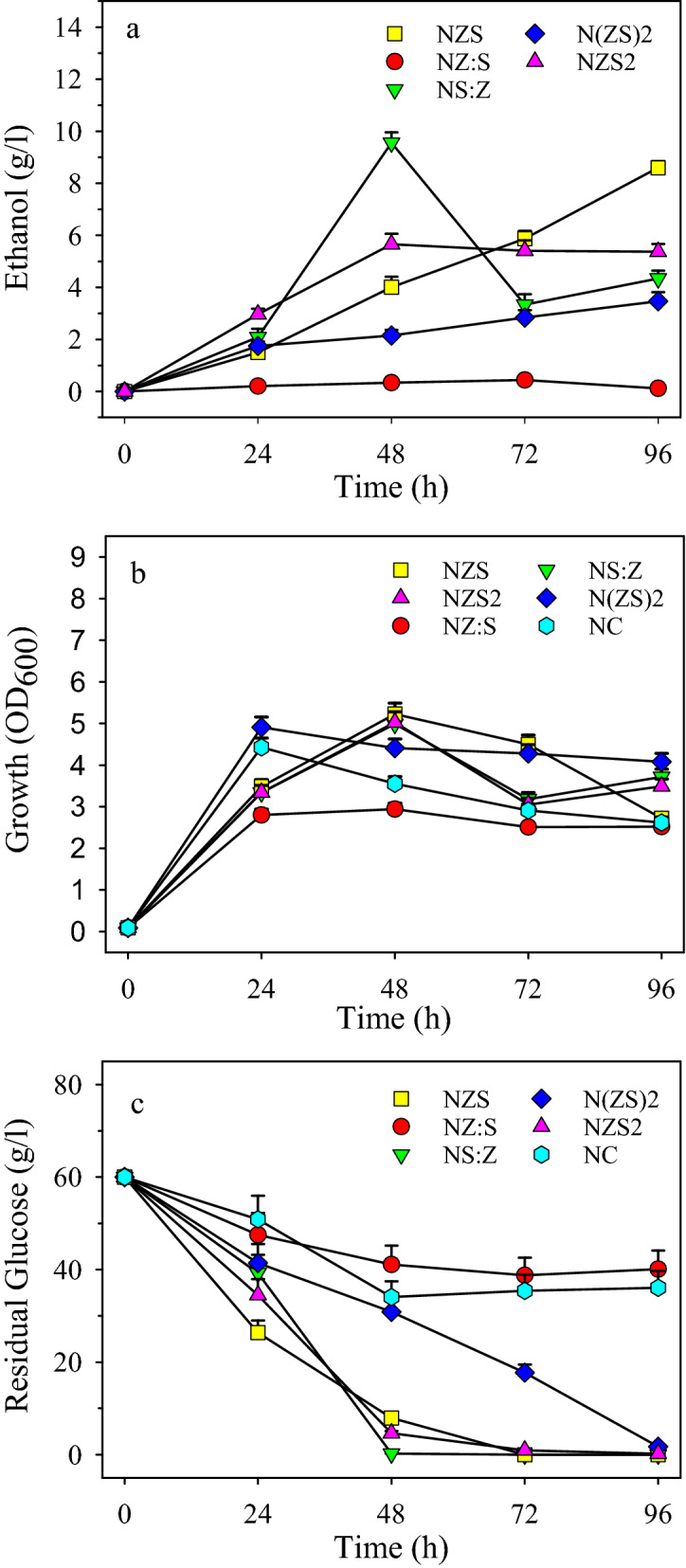
Ethanol production by *B. subtilis* strains harboring different numbers of *pdc*_*Z*_ and *adh*_S_ as well as fusions of the genes. Strains NZS (Δ*ldh*, *pdc*_*Z*_*-adh*_*S*_), N(ZS)2 (Δ*ldh*, (*pdc*_*Z*_*-adh*_*S*_)_2_), NZS2 (Δ*ldh*, *pdc*_*Z*_*-*(*adh*_*S*_)_2_), NZ:S (Δ*ldh*, *pdc*_*Z*_:*adh*_*S*_), and NS:Z (Δ*ldh*, *adh*_*S*_:*pdc*_*Z*_) containing, respectively, one copy of each gene, two copies of each gene, one *pdc*_*Z*_ but two *adh*_*S*_, *adh*_*S*_ fused to *pdc*_*Z*_, and *pdc*_*Z*_ fused to *adh*_*S*_, were cultured with an initial optical density of 0.1 (600 nm) in 2YT mediums supplemented with 60 g/L glucose and incubated at 37 °C, 180 rpm. Samples taken at 24-h intervals were analyzed for: (**a**) ethanol production, (**b**) cell growth, and (**c**) glucose consumption.

The effects of the copy number and relative activity of *pdc*_*Z*_ and *adh*_*S*_ on ethanol production were analyzed using ethanologenic plasmids either containing more than one copy of the genes or having a gene fusion instead of an operon. The strains N(ZS)2, NZS2, NS:Z, NZ:S as well as the control strain NC were cultured in 2YT medium with 60 g/L glucose. The ethanol concentration, growth, and residual glucose were determined at 24 h intervals (Fig. 6). The highest ethanol concentration of 9.6 g/L was detected just after 48 h in the culture medium of strain NS:Z with a productivity of 0.2 g/L/h and a yield of 31%. The next ethanol producer with 8.7 g/L during 96 h of incubation was NZS resulting in productivity of 0.09 g/L/h and a yield of 28%. Although the maximum ethanol production of strain NZS2 was 5.66 g/L, the strain with a productivity of 0.12 g/L/h was revealed to be faster than NZS in ethanol production and glucose consumption during the first 48 h of incubation. Strains NS:Z, NZS2, and NZS consumed about 100%, 92%, and 87% of the initial glucose (60 g/L) during 48 h of incubation of which one third (19 g), one fifth (11.2 g), and one-sixth (8 g) were converted to ethanol, respectively. As for NS:Z and NZS2, the growth peak was temporally corresponding with ethanol production peak and glucose depletion. Therefore, it may be assumed that the relative activity of pyruvate decarboxylase and alcohol dehydrogenase in the strains were well suited to the metabolism of the host resulting in concurrent growth and ethanol production. In contrast, strains NZ:S and N(ZS)2 were adversely affected by the expression of ethanologenic enzymes. As for strain NZ:S, in particular, the growth, ethanol production, and glucose consumption were severely inhibited as a result of the expression of fusion *pdc*_*Z*_*:adh*_*S*_. From the results it could finally be inferred that strain NS:Z was more efficient than other strains in ethanol production, and the lack of enough glucose might have been a major limiting factor for growth and more ethanol production of the strain during the late 48 h of fermentation.

### CBP ethanol production by strain NS:Z using untreated potatoes

**Figure 7:**
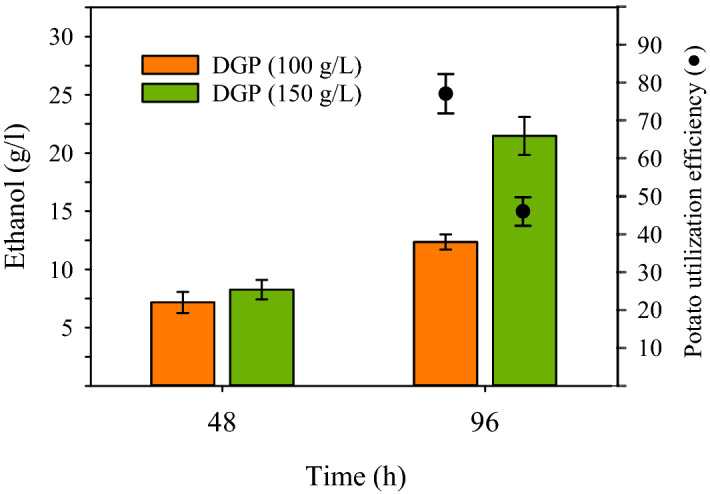
CBP ethanol production by strain NS:Z (Δ*ldh*, *adh*_*S*_:*pdc*_*Z*_). Cultures were conducted in 2YT medium supplemented with 100 and 150 g/L dried ground potatoes (DGP) at 37 °C, 180 rpm under limited aeration conditions. The dots (black filled circle) represent the efficiency of potato utilization (%).

Given the favorable characteristics of NS:Z as an ethanologenic strain, it was evaluated for CBP ethanol production on the untreated potatoes. For this purpose, cultures were conducted in 2YT medium containing 100 g/L and 150 g/L DGP. The results showed that NS:Z was able to grow in such highly viscous mediums, and surprisingly produced high concentrations of ethanol by CBP (Fig. 7). The ethanol production in the mediums with 100 g/L and 150 g/L DGP was not significantly different by 48 h of fermentation, regardless of the initial DGP concentration. However, at the end of fermentation (96 h), the ethanol concentration increased to 12.5 g/L and 21.4 g/L in cultures with 100 g/L and 150 g/L DGP, respectively. In the other words, 73 percent more ethanol was accumulated in the culture with the higher initial DGP concentration. The determination of residual solids in the culture mediums indicated that about 78 g/L and 69 g/L out of the 100 g/L and 150 g/L initial DGP, respectively, have been solubilized over the time course of fermentation. Therefore, the ethanol production yield was estimated at 28% and 54% of the theoretical maximum with 100 g/L and 150 g/L initial DGP, respectively. The impact of strain NS:Z on the solubilization of DGP and the fluidity of the culture medium was inspected by viscosity analysis. While the viscosity of the uninoculated culture medium with 150 g/L DGP remained almost unchanged at 86.25 (P), the viscosity of the culture medium inoculated with NS:Z was significantly decreased to 1.65 (P) during the 96 h fermentation (data not shown). The results showed that NS:Z in a tiny inoculation (initial OD_600 nm_ of 0.1) was able to propagate in the highly viscous medium containing 150 g/L DGP, reducing the viscosity by 52 times as a result of its metabolism and secretion of hydrolytic enzymes.

## Discussion

*Bacillus subtills* can survive on a range of different substrates due to its metabolic diversity and robust systems for the production and secretion of enzymes^16–19^. The bacterium exhibits low nutrient requirements and remarkable tolerance to high concentrations of salt and solvents. These features are of significant importance where bioconversion of low-cost feedstocks to value-added biocommodities is aimed^20,21^. While *S. cerevisiae* and *Z. mobilis,* as the most important ethanologenic organisms, can consume just a few carbohydrates, *B. subtilis* can utilize a diversity of carbon sources. The ability to consume polysaccharides makes *B. subtilis* a promising candidate for the development of ethanologenic strains for bioconversion of plant biomass wastes. In this respect, *B. subtilis* has an elaborate system for the consumption of starch biomass, through which the starch is hydrolyzed by extracellular amylolytic enzymes to release maltose and maltodextrins. Maltose is taken up by the phosphotransferase system (PTS) and hydrolyzed into glucose and glucose-6-P by the cytoplasmic phospho-α-1,4-glucosidase (MalA). Maltodextrins are taken up by a specific ABC transporter without phosphorylation and are degraded into glucose by cooperative actions of a cytoplasmic maltogenic amylase (YvdF), a maltose phosphorylase (YvdK), and a glucosidase (MalL)^22^. Therefore, the use of an ethanologenic *B. subtilis* in CBP system would eliminate the need for gelatinization, liquefaction, and saccharification steps that are currently required for ethanol production from starchy biomass by *S. cerevisiae* (Fig. 1).

The only report of successful development of an ethanologenic *B. subtilis* strain has been published by Romero et al.^6^. They used *Z. mobilis pdc* and *adhB* genes to make a synthetic ethanologenic operon in *B. subtilis*. The operon was inserted into the *B. subtilis* chromosomal *ldh* encoding lactate dehydrogenase, which is responsible for reducing pyruvate to lactate. Consequently, the lactate production as the main fermentation product of *B. subtilis* was disrupted, and instead, the ethanol operon could be expressed under the control of the *ldh* promoter. However, the growth and glucose consumption of the resulting strains were significantly decreased by 70% and 65%, respectively. In this regard, the researchers managed to find out that *B. subtilis* lactate dehydrogenase was able to utilize both NADH and NADPH as cofactor, balancing the cellular concentration of their reduced form. In the ethanologenic Δ*ldh* strain, while the NADH-oxidation activity of lactate dehydrogenase could be fulfilled by *Z. mobilis adhB*, the NADPH oxidation remained unattended, resulting in an unbalanced NADP^+^/NADPH ratio and consequently a lowered growth rate. To tackle the problem the gene coding for *E. coli* transhydrogenase (*udhA*) was inserted by the researchers into the acetolactate synthase gene (*als*) of *B. subtilis*^23^. The transhydrogenase mediates the reciprocal transfer of hydride between NAD(H) and NADP(H), restoring the NADP^+^/NADPH ratio in the absence of lactate dehydrogenase activity. The resulting strain BS37 showed 22% and 59% improvements in the growth rate and glucose consumption, respectively. In addition, the insertional inactivation of *als* resulted in the blockade of butanediol production as a significant rival pathway for ethanol production in *B. subtilis*. Consequently, the ethanol production by the strain was raised to 8.9 g/L in a culture with 20 g/L glucose during 9 days of incubation under nonaerated conditions in minifleakers at 35 °C, 100 rpm, and pH 7^6^. Apart from the study conducted by Romero et al., other attempts for making ethanologenic strains from gram-positive bacteria have not been as successful^5,24–28^. Lactate is the major fermentation product of *B. subtilis* and is produced at substantially higher amounts than acetate and 2,3-butanediol as the next most abundant fermentation products of the bacterium^29^. As in Romero et al. study, the deactivation of the lactate pathway proved in the present study to be crucial for ethanol production by *B. subtilis,* as neither the intrinsic ethanol pathway nor the engineered heterologous pathway was able to elicit noticeable ethanol production in the parental WB600 strain. The resulting Δ*ldh* strain (WBN) was successfully used for ethanol production using a synthetic operon containing *S. cerevisiae* alcohol dehydrogenase (*adhI*), and *Z. mobilis* pyruvate decarboxylase (*pdc*)*.* The strain (NZS) was able to grow and produce ethanol under various aeration conditions. It is noticeable that the problems associated with ethanol production in the Romero et al. study might have been related to the nonaerated culture conditions. In the present study, cultures were conducted under aerated conditions obviating the need for an exogenous transhydrogenase to deal with the unbalance of redox equivalents that may occur with non-aerated cultures in the absence of lactate dehydrogenase activity. The growth of the engineered strains under aerated conditions resulted in high concentrations of ethanol in a shorter period of fermentation. Under such conditions, the ethanologenic strains grew well on potatoes and produced ethanol in CBP at higher concentrations than those obtained with glucose. In this study, the improvement of growth and ethanol production by fusing *Z. mobilis* pyruvate decarboxylase (PDC) to *S. cerevisiae* alcohol dehydrogenase I (ADH) in the ADH-PDC configuration may have resulted from a favorable change in the relative activity of the enzymes. It has been shown that the development of enzyme fusions is an effective approach for making bi- or multi-functional enzymes with improved desirable characteristics^30,31^. The catalytic activity of enzymes is highly likely to be altered in fusions so that it may be increased or decreased for both enzymes or increased for one but decreased for the other enzyme. In addition, the orientation of domains in a fusion enzyme may significantly affect the enzyme properties^32,33^.

Despite attempts towards CBP ethanol production, there are only a few successful reports on direct bioconversion of plant biomass into ethanol by a single organism. In a study, *Okamoto* et al. managed to produce 9.8 g/L ethanol from 20 g/L commercial corn starch by white-rot basidiomycete *Trametes versicolor*. The yield obtained was significant but the required 7-day precultures on nutrient-rich medium was a drawback to cost-effective ethanol production^34^. Likewise, *Tanimura* et al. used a natural isolate of *Scheffersomyces shehatae* for 9.87 g/L ethanol production from 100 g/L starch in 10 days; however, the yield was lower than that obtained by *Trametes versicolor*^35^. In a study by Hossain et al., the researchers used *Wickerhamia* sp. for CBP ethanol production from potato peel waste (PPW). Thereby, they produced 7.44 g/L ethanol from 40 g/L PPW after 96 h fermentation that could be raised to 21.7 g/L after statistical optimization of culture medium and supplementation of 25 g/L malt extract^36^. Although the high amounts of malt extract consumption represent a disadvantage for economical bioethanol production, their study is interesting in terms of direct bioconversion of industrial potato peel waste into biofuel. In a more recent study by Bibra et al., *Geobacillus thermoglucosidasius* was used for ethanol production from food waste in a single pot at 60 °C. They managed to produce 3.03 g/L ethanol from 10% (w/v) of untreated food waste in serum bottles during 96 h, and 13 g/L in 1L reactor during 168 h. The sequential cultivation of *Geobacillus thermoglucosidasius* and *Thermoanaerobacter ethanolicus* resulted in an increase to 16.1 g/L during 128 h in 1 L bioreactor, and 18.4 g/L during 120 h in 40 L bioreactor with 20% food waste (w/v)^37^. Given the prominent features of *S. cerevisiae* in ethanolic fermentation, this microorganism has been subjected to various genetic manipulation strategies to meet the industrial requirements of CBP ethanol production. In a successful study, Cripwell et al. used the α-amylase and glucoamylase coding genes of *Talaromyces emersonii* for engineering amylolytic *S. cerevisiae* using industrial strains Ethanol Red and M2n as parental cells^38^. The resulting strains, ER T12 and M2n T1, were evaluated in a separate study by Myburgh et al. for CBP ethanol production from broken rice. The results were quite encouraging with the achievement of the maximum expected value of ethanol production^39^. However, with the diversity of feedstocks in terms of complexity and components, the development of custom-designed CBP ethanol producers is quite imperative. For this purpose, research on various types of microorganisms including fungal and bacterial strains would be necessary.

## Conclusions

*Bacillus subtills* exhibits important features to be used for bioethanol production from biomass. In this study, several ethanologenic strains of *B. subtilis* were developed and evaluated for aerobic bioethanol production. The results obtained in this study suggest that *B. subtilis* shows potentials to be developed into a significant producer of CBP ethanol from low-cost agricultural wastes. In this context, the capability of *B. subtilis* to utilize polymeric carbohydrates may be extended by the expression of synthetic genes encoding select enzymes required for the efficient digestion of complex plant biomass.
